# Outbreak of *Pseudomonas aeruginosa* perichondritis associated with ear piercings and a contaminated water system

**DOI:** 10.1017/S0950268824001572

**Published:** 2025-01-10

**Authors:** Claire E. Brown, Derren Ready, Caroline Willis, Ben Sims, Nick Young, Elizabeth Sheridan, Helen Osbourne, Louise Jones, Yvette Landy, Naomi Long, Amy Walkden, Jane F. Turton, Karren Staniforth, Ginny Moore, Simon Parks, Patricia Barkoci, Sarah Bird

**Affiliations:** 1 Health Protection Operations, South West, UK Health Security Agency, Bristol, UK; 2 NIHR Health Protection Research Unit in Behavioural Science and Evaluation, University of Bristol, Bristol, UK; 3 Food Water and Environmental Microbiology Laboratory Porton, UK Health Security Agency, Salisbury, UK; 4 Department of Microbiology, University Hospitals Dorset, Dorset, UK; 5 Environmental Health Team, Bournemouth, Christchurch and Poole Council, Dorset, UK; 6 HCAI, Fungal, AMR, AMU and Sepsis Division, UK Health Security Agency, London, UK; 7 Diagnostics and Pathogen Characterisation, UK Health Security Agency, Salisbury, UK

**Keywords:** ear piercing, infection prevention and control, *P. aeruginosa*, perichondritis, water system

## Abstract

In September 2023, the UK Health Security Agency’s (UKHSA) South West Health Protection Team received notification of patients with *Pseudomonas aeruginosa* perichondritis. All five cases had attended the same cosmetic piercing studio and a multi-disciplinary outbreak control investigation was subsequently initiated. An additional five cases attending the same studio were found. Seven of the ten cases had isolates available for Variable Number Tandem Repeat (VNTR) typing at the UKHSA national reference laboratory. Clinical and environmental *P. aeruginosa* isolates from the patients, handwash sink, tap water and throughout the wall-mounted point-of-use water heater (including outlet water) were indistinguishable by VNTR typing (11,6,2,2,1,3,6,3,11). No additional cases were identified after control measures were implemented, which included replacing the sink and point-of-use heater.

The lack of specific recommendations to control for *P. aeruginosa* within Council-adopted ear-piercing byelaws or national guidance means that a cosmetic piercing artist could inadvertently overlook the risks from this bacterial pathogen despite every intention to comply with the law and follow industry best practice advice. Clinicians, Environmental Health Officers and public health professionals should remain alert for single cases of *Pseudomonas* perichondritis infections associated with piercings and have a low threshold for notification to local health protection teams.

## Introduction

*P. aeruginosa* is a Gram-negative, opportunistic, bacterial pathogen commonly found in wet environments with the ability to form biofilms in water systems including contamination of taps, flow straighteners, sinks and pipes [1]. This bacterium can colonise and infect the human body and is known to cause a range of opportunistic infections in susceptible people, including infections of skin and soft tissues, bloodstream, respiratory and urinary infections [2].

*P. aeruginosa* is a recognised cause of sporadic piercing-related infections, generally occurring 2–4 weeks after the piercing has taken place. Transmission typically occurs through direct contact of the piercing site with water or aftercare products contaminated with *P. aeruginosa* [3, 4]. Infections have previously been associated with swimming in fresh/pool water and poor hygiene [3, 4]. *P. aeruginosa* is the most common causative agent of perichondritis [5, 6] and piercing-related perichondritis is becoming more likely as high ear piercings (defined as involving the superior third of the pinna cartilage) have grown in popularity [7]. High ear piercing carries an increased risk of infection and complications because the area has a low blood supply. If left untreated, perichondritis can result in invasive infections or permanent deformity of the ear [3, 5].

There are few reports of outbreaks of *P. aeruginosa* associated with piercings. These have identified high ear piercing, contaminated aftercare solutions, newly trained staff and discount events to encourage higher demand, as potential risk factors [1, 4]. As *Pseudomonas* species is not a notifiable causative agent under the Health Protection (Notification) Regulations 2010 [8] burden of infections related to ear piercing is difficult to determine.

In September 2023, UK Health Security Agency (UKHSA) South West was notified by a local hospital of a cluster of five cases of perichondritis that had been treated by the ENT department following ear piercings undertaken at the same piercing venue. At the time, *P. aeruginosa* had been cultured from specimens collected from four of the cases. All cases required admission to the hospital for at least 48 h with intravenous antibiotics administered and three cases required surgical incision and drainage. A multi-agency outbreak control team (OCT) was convened and led by the UKHSA South West Health Protection Team (HPT) to identify the source and extent of the outbreak and to support the implementation of control measures to prevent further infections. Investigations focused on identifying if the causative strain was common to all cases and whether there were any common source exposures, which included an investigation of the piercing venue environment. We describe the investigation and control measures taken for this outbreak.

## Methods

### Case finding and case definitions

An alert was sent to local acute clinicians and primary care providers highlighting the possibility of *P. aeruginosa* infection in patients presenting with a skin infection at the site of a recent piercing. They were asked to seek advice on appropriate management of such infections and to notify confirmed or suspected cases to infection control teams (in acute settings) and the local health protection team. Using client lists provided by the venue under investigation, a “warn and inform” letter was sent to all individuals who had any piercing performed at the venue since 01.08.2023 (100 people). The letter was sent to raise awareness of signs and symptoms of piercing infection and to ask anyone who had experienced an infection to contact the HPT, to assist in case finding and epidemiological investigation.

For the purpose of the outbreak investigation, cases were defined as a confirmed outbreak case if a person had a laboratory culture-confirmed *P. aeruginosa* infection with the outbreak strain / Variable Number Tandem Repeat (VNTR) type 11,6,2,2,1,3,6,3,11; following a body-piercing (any part of the body) at the venue under investigation from 1st August 2023 onwards; a probable case was as confirmed, but with no strain VNTR typing being available.

### Epidemiological methods

All individuals identified as having a piercing infection following piercing at the venue under investigation were interviewed using a bespoke online trawling questionnaire to gather information including exposures and clinical presentation. This included information about the piercing including body site, processes used in the venue, aftercare advice given and practice, product provided/used, symptoms and if medical care was sought.

### Clinical microbiological methods

Samples collected from infected piercing sites were cultured to recover *P. aeruginosa* and sent to the national Antimicrobial Resistance and Healthcare Acquired Infections (AMRHAI) reference unit for confirmation of identity and further typing. Typing was carried out using Variable Number Tandem Repeat (VNTR) analysis at nine loci as previously described [9, 10].

### Environmental investigations

The ear-piercing service was being provided by a self-employed cosmetic piercer who had previously registered with the local Council in accordance with the provisions of the Local Government (Miscellaneous Provisions) Act 1982. Upon first registration, the business had been proactively visited by an Environmental Health Officer and there had been no cause for concern with respect to compliance with the locally adopted Byelaws for ear-piercing and cosmetic piercing. With the exception of the circumstances of the outbreak, the Council had not received any recent complaints about hygiene or safety at the studio or the ear-piercing activity specifically.

All cosmetic piercing was carried out in a separate small room to the tattooing area and this was at the rear of the premises. Inside the room was a portable trolley that was used to make the equipment readily accessible during the piercing procedure. The room had a single handwash basin with hot water provided via a wall-mounted point-of-use water heater. A foot-operated bin was used for clinical waste and a sharps bin was available for all sharps waste. Reusable equipment was placed on one side for cleaning before the next use. A small autoclave used to sterilise jewellery was situated in the tattoo area, however, this remained accessible to the cosmetic piercing artist.

The venue was visited the day after notification (day 1) by Environmental Health to review procedures related to piercing, and 16 environmental samples were taken from the taps and the sink. Aftercare solutions, soap, disinfectants, alcohol wipes, environmental cleaning solutions and hot and cold water samples were also collected. Follow-up environmental and water samples from the ear-piercing room were taken 18 days later, after the sink and wall-mounted point-of-use water heater had been replaced. As part of the remedial work, the wall-mounted, point-of-use, water heater from the ear-piercing room was removed and replaced with a new heater. The original heater was removed from the premises and transported to UKHSA where it was dismantled and tested.

Sampled products were transported to the UKHSA Food Water and Environmental Microbiology (FW&E) Laboratory and tested using a standard method. For soaps and solutions, an aliquot of 25 g (or all of the available volume if less than 25 g) was homogenised with sufficient Buffered Peptone Water (BPW) to prepare a 10^−1^ dilution. Swabs were immersed in 100 ml of BPW and agitated to disperse any micro-organisms. A portion (0.5 ml) of this suspension was inoculated directly onto *Pseudomonas* CN Selective Agar (PCN) plates and incubated at 37 °C for 48 h. The remaining volume was incubated at 30 °C for 24 h after which time, a 10 μL portion was sub-cultured to a PCN plate, which was then incubated at 37 °C for 48 h. For water samples, a 100 ml aliquot was filtered through a cellulose ester membrane (0.45 μm pore size) using negative pressure. Filter membranes were placed onto PCN plates and incubated as above. Presumptive *Pseudomonas* colonies (blue/green or yellow/green and oxidase positive) were further identified using a MALDI-ToF instrument (Bruker). Representative isolates from each sample giving colony identifications as *P. aeruginosa* were sent to the national UKHSA reference unit for confirmation of identity and VNTR typing, as previously described [9, 10].

## Results

**Figure 1.**
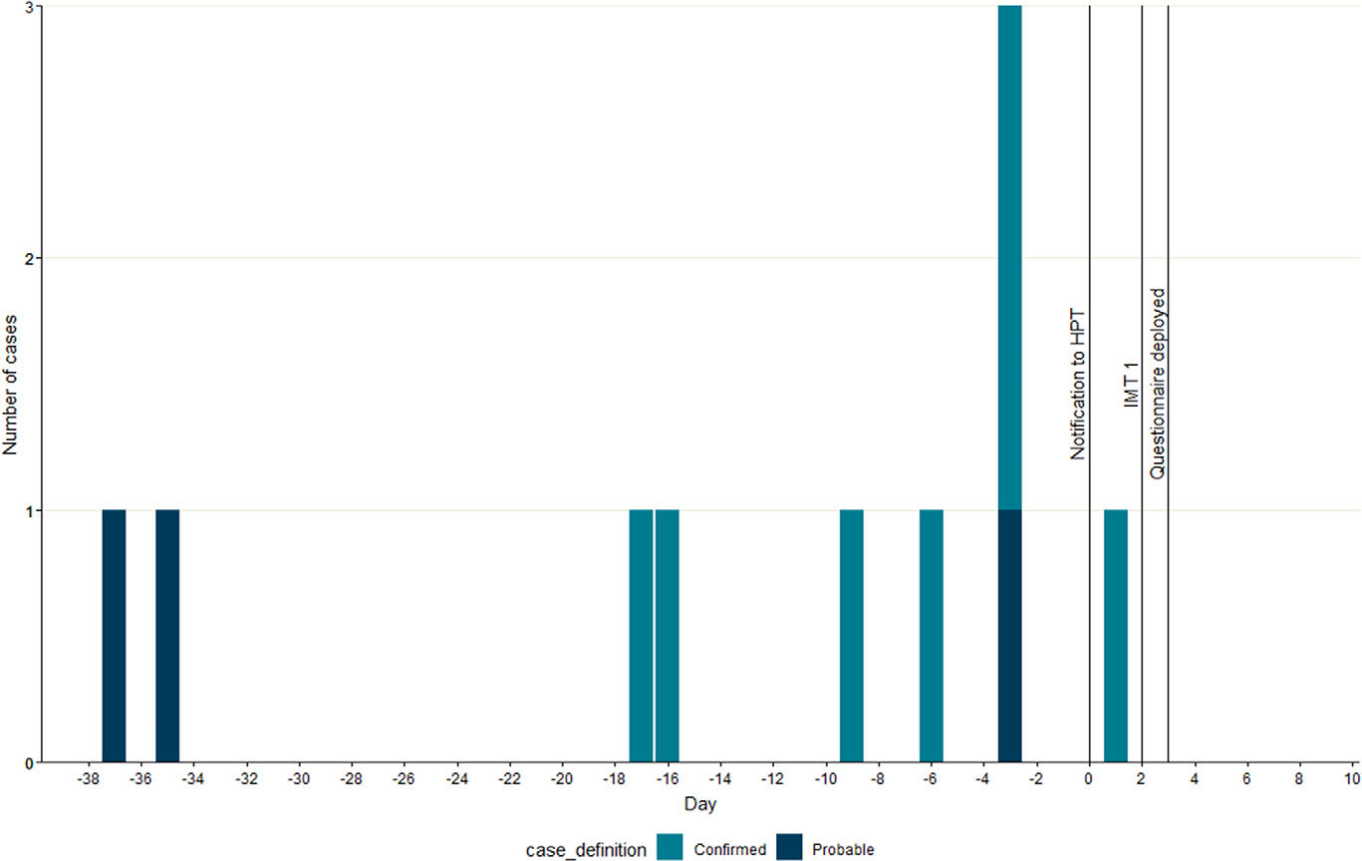
Epidemic Curve: day of onset of confirmed and probable cases (n = 10).

Seven confirmed and three probable cases were identified, with dates of piercing at the venue between 37 days before notification and the day of notification (Figure 1). All cases were female, with a median age of 26 years (range: 14–49 years); seven cases required admission to the hospital, and all reported attending secondary care because of their infection. Questionnaires were completed with 8/10 cases (5 confirmed and 3 probable). For the 8 cases completing the questionnaire, the median age was 26 years (range: 17–49 years). All cases reported cartilage ear piercings (4/8; 50% Helix, 3/8; 38%Tragus, 1/8; 13% antihelix). The median number of days from piercing to first sign of infection was reported to be 3 days (Range: 0–4 days). All cases reported that a needle was used to perform the piercing. None of the cases reported the use of a spray or solution during or immediately after the piercing process, five cases mentioned the use of a wipe on the ear area. Three cases reported purchasing an aftercare solution/spray from the venue to use at home. A range of other aftercare options were reported by cases including homemade saline solution/spray (6/8; 75%), solution/spray purchased elsewhere (2/8; 25%), and antibacterial purchased elsewhere (1/8; 13%). In addition, two cases (25%) reported using an antibacterial cream provided by their primary care General Practitioner (GP). Cases commonly reported accessing healthcare services for their infection, including GP services (8/8; 100%), telephone advice via NHS 111 (7/8; 88%), ear, nose and throat (7/8, 88%), accident and emergency (6/8; 75%) and five cases reported admission to the hospital (63%).

## Microbiology

**Figure 2.**
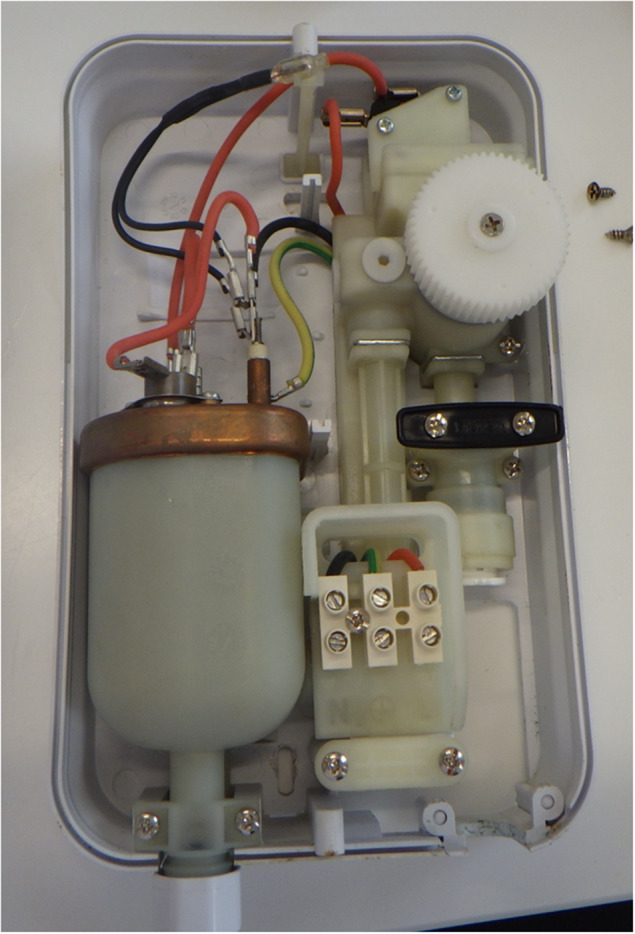
Wall-mounted water-heater unit.

Bacterial isolates recovered from three of the cases were not available for VNTR typing as they had been discarded. Isolates from all 7 patients for which isolates were available and from environmental samples (n = 7; Table 1) collected from sinks, cold water taps and the wall-mounted, point-of-use, water heater, all shared a unique VNTR profile of 11,6,2,2,1,3,6,3,11. A general profile of 11,6,2,2,1,3,x,x,x (where x is variable) corresponds to the ‘clone C’ lineage, which is commonly found in the environment and associated with human infections [9]. Follow-up samples of water from the ear-piercing room 18 days later, after remedial action, were negative for *P. aeruginosa.* As part of the remedial work, the wall-mounted point-of-use water heater (Figure 2) from the ear-piercing room was removed and dismantled and individual components were tested for the presence of *P. aeruginosa.* Heavy colonisation with *P. aeruginosa* was detected in multiple components (valves, O-rings and tubing) before and after the heating element within the unit, although no growth was observed from the heater itself.

**Table 1.** *Pseudomonas aeruginosa* results for swabs and water samples from the ear-piercing and tattoo rooms in the premises used by patients

Date of sampling	Location	Sampling point	Ps. aeruginosa result	Outbreak strain
Day 1	Ear-piercing room	Swab of point-of-use hot water unit	Detected	Yes
		Swab of point of use hot tap	Not detected	–
		Swab of the tap handle	Not detected	–
		Cold water	2 cfu in 100 ml	Yes
		Water from the autoclave reservoir	Not detected in 100 ml	–
Day 2	Ear-piercing room	Swab of plug-hole	Detected	Yes
		Swab of cold tap	Detected	Yes
		Swab of wash basin	Detected	Yes
		Swab of disinfectant bottle	Not detected	-
		Point of use hot water	>100 cfu in 100 ml	Yes
		Cold water	1 cfu in 100 ml	Yes
		Aftercare solution	Not detected	–
		Antimicrobial liquid soap	Not detected	–
		Enzymatic instrument cleaner	Not detected	–
		Topical disinfectant solution	Not detected	–
		Alcohol wipes	Not detected	–
Day 19	Ear-piercing room	Cold water tap		
		-pre-flush	Not detected in 100 ml	–
		-post flush	Not detected in 100 ml	–
		Point of use hot water tap		
		-pre-flush	Not detected in 100 ml	–
		-post flush	Not detected in 100 ml	–
		Swab of the wash basin	Not detected	–
		Swab of plug hole	Not detected	–

### Environmental investigations

Piercing was undertaken either by appointment or on a walk-in basis. Every client was required to complete a consent form, and this helped the investigators ascertain the number of people who had received a piercing.

The procedures for preventing the risk of infection were determined by interviewing the piercer. Reusable body-piercing equipment, such as clamps, were initially hand washed in the utensil wash sink and then soaked in metal lidded trays for 30 min in a bactericidal multi-enzyme detergent concentrate diluted with water from the washbasin in accordance with the manufacturer’s instructions. The equipment was then rinsed, air-dried, and placed in autoclave bags and then autoclaved for 45 min. The sterilised equipment was subsequently stored in its bag ready for use for a maximum of 3–4 months.

All surfaces in the piercing room were cleaned with a cleaning and disinfection solution (Chemgene) using disposable paper towels that were disposed of in a foot-operated bin. Hands were washed with a pre-prepared antimicrobial hand and skin cleanser (Opti-Scrub) and dried with disposable paper towels. Gloves were then worn. Equipment used for the piercing was set out in advance including clamps, needle, guide pin, gloves, jewellery, pre-injection swab, spare paper towel for wiping excess petroleum jelly or bleeding, ink (gentian violet), disposable ink pot and a toothpick.

The client’s skin was cleaned with a 70% alcohol pre-injection swab. A toothpick is used to mark a dot for the piercing site on the skin using ink from a disposable ink pot. The toothpick is then discarded.

A single-use needle is used to pierce the skin and other equipment is unpackaged from the sterilised autoclave bags as required. A pre-made aftercare product is offered for post-piercing care.

### Control measures

The existing Council-adopted byelaws for ear-piercing and cosmetic piercing refer in general terms to the cleansing and sterilisation of equipment and require, among other things, an adequate supply of hot and cold water. There are no specific references within the byelaws to control for *P. aeruginosa* in the water supply. Similarly, the CIEH Tattooing and body-piercing guidance toolkit [11] includes comprehensive guidance on many aspects of health and safety and infection control, however, the emphasis is on the control of blood-borne viral infections. The lack of specific byelaws or guidance, consequently, means that a cosmetic piercing artist could inadvertently overlook *P. aeruginosa* as a risk despite having the best of intentions to follow industry best practices.

Once the problem had been brought to the attention of the business in question, the piercing artist voluntarily ceased higher ear-piercing activities on day 2 and all piercing activities on day 4 while remedial action was undertaken.

A bespoke letter was drafted and sent by the IMT to the business to help mitigate the risk, which included the use of alcohol hand gel post-handwashing. This provided details of the investigation findings to date, information about *P. aeruginosa* and detailed steps that could be taken to reduce the risk to future clients.

## Discussion

This investigation found strong environmental, microbiological and descriptive epidemiological evidence that an outbreak of *P. aeruginosa* infections following ear piercings carried out in the South West was associated with a single venue. All cases were in females, which concurs with the findings of a systematic review which reported that post-piercing perichondritis was most commonly seen in adolescent and young adult females [12]. According to the review patients typically delay seeking medical care for approximately one week following the initial onset of symptoms. This might suggest that the verbal and written aftercare advice clients’ receive following their piercing could be improved to further encourage healthcare-seeking behaviour when their piercing is not healing as expected. In this outbreak, one additional case came forward because of the warning and inform letter, which demonstrated an added case ascertainment value to sending these letters.

The median number of days between the piercing date and onset of first symptoms was 3 days which supported the hypothesis that these infections occurred around the time of the piercing procedure rather than resulting from exposure to a contaminated aftercare solution. In previous studies, in which perichondritis outbreaks were linked to *P. aeruginosa*-contaminated aftercare solutions or sprays, longer time scales were seen before symptoms appeared, typically being 14–15 days [4, 13]. In this incident, the rapid deployment of a bespoke trawling questionnaire allowed the Incident Management Team to quickly rule out any common aftercare usage between the cases and focus controls at the venue.

Environmental swabs and water samples from the venue recovered isolates of *P. aeruginosa* that were indistinguishable by VNTR typing from the seven confirmed human cases. The use of VNTR typing was key to this investigation in identifying that a common type was shared between cases and environmental samples taken from the business. Whole genome sequencing (WGS) provides a high level of strain characterisation and discrimination [13], as VNTR is the accredited methodology for *Pseudomonas* typing currently used by the UKHSA reference laboratory this allowed comparison with an existing database of over 40,000 previously typed isolates. Although 19 VNTR loci are described for *Pseudomonas*, for routine, rapid typing, a nine-locus scheme has previously been shown to offer sufficient discrimination between types [10]. Interestingly, environmental isolates from the water, sink, and throughout the wall-mounted point-of-use water heater suggested that contamination of the water system allowed a reservoir to persist in the environment. The persistence of *P. aeruginosa* in water systems is well recognised as a risk in the healthcare environment [14], and there is a requirement in the UK for water in augmented care areas of hospitals to be monitored for this organism (HTM 04-01) and for remedial actions to be put in place where high levels are detected [15]. However, there are no similar requirements for monitoring and control of hot and cold water supplies outside of the hospital environment. It is evident that the invasive nature of ear-piercing provides a portal of entry for this pathogen and that it would be advantageous to update the existing toolkit to facilitate risk reduction strategies. It has previously been shown that the piercing method does not impact on risk of perichonditis and prevention of post-piercing perichonditis should focus on hygiene and aftercare [16].

Post-remedial action testing at day 19 did not recover *P. aeruginosa* from the water systems and no additional cases were identified after control measures were implemented and remediation of the venue was completed. In a similar outbreak in North West England in 2016, indistinguishable *P. aeruginosa* types were isolated from cases and environmental samples taken from taps and sinks following an ear-piercing event [1], however, this outbreak was also associated with an inexperienced practitioner. Piercings can be carried out by untrained or inexperienced individuals, who may have little to no knowledge of appropriate aseptic techniques resulting in suboptimal hygiene and decontamination processes [1, 3]. Therefore, the availability of clear guidelines to reduce the risk of post-piercing bacterial infections is key, especially, in conjunction with proactive and close working of businesses with Environmental Health teams [1].

## Recommendations

Recommendations to piercers should focus on education, hygiene and aftercare;

- Good record-keeping to support case ascertainment and warn and inform communication.
- Use of single-use sterile products where possible.
- Ensure verbal and written advice is given to customers to highlight the risk of high ear piercings and encourage clients’ healthcare-seeking behaviour. Aftercare leaflets are available within the CIEH toolkit.
- Ensure training of piercing practitioners in appropriate aseptic techniques
- Ensure regular maintenance of water systems and heaters

Recommendations to clinicians and public health professionals;

- Improve case ascertainment using letters to health professionals and customers.
- Trawling questionnaires can be used to rapidly identify common exposures among cases.
- In outbreak settings water samples should be collected from sinks traps, water heaters and water outlets.
- Environmental Health Practitioners to foster close working relationships with local venues.
- Guidance to tattooists and piercers could be updated to cover the risk of both *Pseudomonas* infections and contaminated water systems.

## Conclusion

Compared to piercing of the ear lobe, high ear (cartilage) piercings are associated with a greater risk of complications which can be more difficult to treat [3]. Accurate and prompt diagnosis of *Pseudomonas* perichondritis is key as it requires treatment with appropriate antimicrobial therapy [7]. As *P. aeruginosa* perichondritis infection can lead to abscess formation, loss of cartilage and complications requiring surgical intervention, antibiotic therapy, drainage, and debridement, resulting in potentially lifelong disfigurement, clinicians should remain alert for cases of *Pseudomonas* perichondritis infections associated with piercings. Whilst all piercing procedures carry a risk of infection it is unusual to see a cluster of inpatient admissions of this severity in a short period. Rapid public health action was likely to have prevented further cases as no additional cases were seen after controls and remediation were put in place.

## Data Availability

This manuscript describes a limited series of cases, and as such there is no broader dataset for release. Any queries regarding the data can be directed to the authors.
